# *Patrinia villosa* (Thunb.) Juss alleviates CCL_4_-induced acute liver injury by restoring bile acid levels and inhibiting apoptosis/autophagy

**DOI:** 10.3389/fphar.2024.1409971

**Published:** 2024-05-22

**Authors:** Ji-Feng Ye, Wei Liu, Qishu Hou, Shu-Qi Bai, Zheng Xiang, Jiaqi Wang, Liman Qiao

**Affiliations:** ^1^ Department of Pharmacy, The Second Affiliated Hospital and Yuying Children’s Hospital of Wenzhou Medical University, Wenzhou, China; ^2^ School of Pharmaceutical Science, Liaoning University, Shenyang, China; ^3^ Shenyang Key Laboratory for Causes and Drug Discovery of Chronic Diseases, Liaoning University, Shenyang, China; ^4^ Liaoning Inspection, Examination and Certification Centre, Liaoning Province Product Quality Supervision and Inspection Institute, Shenyang, China

**Keywords:** bile acids, liver injury, *P. villosa*, autophagy, apoptosis

## Abstract

**Background:**

*Patrinia villosa* (Thunb.) Juss is one of the plant resources of the famous traditional Chinese medicine “Bai jiang cao (herba patriniae),” and it is considered to function at the liver meridian, thereby treating diseases of the liver as demonstrated by the traditional theory of TCM. Unfortunately, the therapeutic mechanism of the whole plant of PV is so far unknown.

**Method:**

UPLC QTOF-MS/MS was used to analyze the profile of PV. Male Sprague–Dawley rats were categorized into five groups, and PV groups (125 and 375 mg/kg) were administered by oral gavage for seven consecutive days. The model of liver injury was induced by intraperitoneal injection of 40% CCl_4_ oil solution. H&E staining was performed for histological evaluation. The ELISA method was used to assess the serum level of ALT, AST, and T-BIL. Serum and liver bile acid (BA) profiling was analyzed by LC-MS/MS. TUNEL-stained liver sections were used to monitor apoptosis caused by CCl_4_. HepG2 cells were used to detect autophagy caused by CCl_4_.

**Results:**

A total of 16 compounds were identified from the 70% methanol extract of PV. PV (125 and 375 mg/kg) could reverse the ectopic overexpression of AST, ALT, and T-BIL caused by CCl_4_ administration. H&E staining indicated that PV (125 and 375 mg/kg) could reduce the infiltration of inflammatory cells and restore liver tissue and hepatocyte structures. Six bile acids, including DCA, HDCA, GCA, TCA, TCDCA, and TUDCA, were significantly altered both in the serum and liver tissue after CCl_4_ administration, and the level of all these six bile acids was restored by PV treatment. Moreover, PV inhibited apoptosis caused by CCl_4_ stimulation in liver tissue and suppressed autophagy in HepG2 cells treated with CCl_4_.

**Conclusion:**

The results in this paper for the first time reveal the alteration of the bile acid profile in CCl_4_-induced liver injury and demonstrate that inhibiting apoptosis and autophagy was involved in *P. villosa*-elicited liver protection, providing a scientific basis for the clinical utilization of *P. villosa* as a natural hepatic protective agent.

## 1 Introduction

The whole plant of *Patrinia villosa* (Thunb.) Juss is one of the plant resources of the famous traditional Chinese medicine (TCM) “Bai jiang cao (herba patriniae),” possessing the function of eliminating heat/toxic materials and removing blood to relieve pain according to the theory of TCM. Herba patriniae boasts a wealth of beneficial compounds, with 233 of them identified in total. Among these are triterpenoid saponins, flavonoids, organic acids, iridoids, and volatiles. Notably, *Patrinia scabiosifolia*, the other plant source of patriniae, is particularly rich in triterpenoid saponins and volatiles, whereas *P. villosa* contains a higher concentration of flavonoids. Despite these differences, both source species of patriniae offer similar pharmacological benefits, including anti-cancer, anti-inflammatory, antioxidant, antimicrobial, sedative, and hypnotic effects. However, it is worth noting that there are no reports on antipruritic, proangiogenic, and anti-diarrheal effects for *P*. *scabiosifolia*, and there are no studies on anti-diabetic effects of *P. villosa*. In general, it is safe to consume patriniae at clinical doses, as it is non-toxic. However, mild side effects such as temporary leukopenia, dizziness, and nausea may occur with excessive and large doses (Gong, et al., 2021).

*P. villosa* is considered to function at the stomach, large intestine, and liver meridians, thereby treating diseases of these organs as demonstrated by the traditional theory of TCM. Data mining has shown that *P. villosa* is frequently used in compound prescriptions for treating liver diseases such as liver chronic hepatitis B (Gao, 2005; Ke, et al., 2022). Previous studies indicated that *P. villosa* has a protective effect on acute liver injury (Qiao et al., 2022). However, the chemical composition, effect, and mechanism of *P. villosa* modulating the levels of bile acids in acute liver injury are known.

Acute liver failure (ALF) is a serious condition that can have various causes. However, drug-induced liver damage is the most frequent cause in developed nations, whereas viral hepatitis is more prevalent globally. Despite the different causes, ALF has similar clinical characteristics. It is imperative to stay informed about the potential causes and symptoms of ALF to prevent and treat it effectively (Vasques et al., 2022). Nowadays, it has been generally acknowledged that herbal medicine can alleviate acute liver injury through multiple mechanisms (Tang et al., 2017; Yuan, et al., 2018).

Over the past decade, an increasing amount of evidence has suggested that bile acids (cholesterol catabolism end products), beyond their role in lipid digestion and absorption, also act as signaling molecules involved in inflammatory responses related to cholestatic liver injury (Li et al., 2017), but if bile acids are involved in acute liver injury, especially in the CCl_4_-induced one, is yet unknown.

In this paper, we for the first time reveal the profile of bile acids in acute liver injury caused by CCl_4_ and demonstrate the inhibition of apoptosis and autophagy in liver cells by *P. villosa* a apoptosis and autophagy are quite important in liver cell function and acute liver injury (Schwabe and Luedde, 2018; Liu et al., 2022; Liu et al., 2023), providing a scientific basis for the further utilization of *P. villosa* as a natural protective agent against liver injury.

## 2 Materials and methods

### 2.1 Materials

*Patrinia villosa* (Thunb.) Juss was obtained from Xinqi Traditional Chinese Medicine Pellets Co., Ltd (Hebei, China), and was identified by Dr. Jian Wu from Harbin University of Commerce. The voucher specimen (No. PVJ20190311) was deposited at the Liaoning University. Bile acids were obtained from Dalian Meilun Biotech Co., Ltd (Dalian, China). All the antibodies were purchased from Santa Cruz Biotechnology, United States of America.

### 2.2 Preparation of the PV extract

A total of 3.5 kg of PV was refluxed with 70% (v/v) methanol for 1 h and evaporated with reduced pressure to create the PV sample.

UPLC-QTOF MS/MS (Agilent 6550 Q-TOF-MS) was used to analyze the major chemical compounds of the PV extract. The mobile phase comprised water (A) and acetonitrile (B). A gradient program was used as follows: 0–6 min, 5%–12% B; 6–18 min, 12%–50% B; 18–20 min, 50%–100% B; 20–22 min, 100% B; 22–22.01 min, 100%–5% B; and 22.01–25 min, 5% B. The flow rate was 0.8 mL/min, the sample injection volume was 1 μL, and the column temperature was 30°C.

### 2.3 Animal experiments

Male Sprague–Dawley rats were purchased from Liaoning Changsheng Biotechnology Co., Ltd. All the animal experiments were conducted following the Guide for the Care and Use of Laboratory Animals approved by the Ethics Committee of Liaoning University (permission no. 20190513101). Rats of 200 ± 20 g weight were categorized into five groups (n = 6): normal, silymarin, CCl_4_, and CCl_4_+PV groups (shown as PV groups for the following context) with two dosages (125 and 375 mg/kg). After 2 weeks of adaption, rats in silymarin, PV, and CCl_4_ groups were injected with 40% CCl_4_ (v/v, olive oil, 2 mL/kg) intraperitoneally. Rats in PV groups were subjected to intragastric administration one time/day for seven consecutive days, and those in normal and CCl_4_ groups were treated the same way with an equal volume of saline. Intraperitoneal anesthetization with sodium pentobarbital was performed on the seventh day.

### 2.4 Bile acid profile

UPLC-QTRAP MS (Waters Acquity UPLC system with AB Sciex-4000 QTRAP MS and Agilent ZORBAX SB-C18 column) was used to test the contents of bile acids (column (3.5 μm, 2.1 × 100 mm), flow rate: 0.4 mL/min, temperature: 25°C, and mobile phase: methanol–water (10:90–90:10)). The linear equation, linear regression equation, *R* ^2^, and linear interval for each bile acid are provided in supporting data.

### 2.5 Cell culture

To study the autophagy in the human hepatoma HepG2 cell line, we obtained the cells from the Cell Bank of Type Culture Collection of the Chinese Academy of Sciences (Shanghai, China); then the cells were grown in Minimum Essential Medium (MEM), supplemented with 10% (v/v) heat-inactivated fetal calf serum (FCS), 110 mg/L sodium pyruvate, 100 units/mL penicillin, and 100 μg/mL streptomycin, and placed in a humidified atmosphere of 95% air and 5% CO_2_ at 37°C.

### 2.6 TUNEL assay

The liver tissue’s cell apoptosis was identified using a TUNEL Assay Kit (terminal deoxynucleotidyl transferase-mediated dUTP nick-end labeling) following the manufacturer’s protocol from Vazyme Biotech Co., Ltd, Nanjing, China. After TUNEL labeling, the liver sections were counterstained with 4′-6-diamidino-2-phenylindole to label the nuclei. Images were observed under a fluorescence microscope from Leica Microsystems, Wetzlar, Germany.

### 2.7 Statistics analysis

Data are expressed as mean ± SEM with one-way analysis of variance (ANOVA); *p*-value <0.05 was considered statistically significance.

## 3 Results

### 3.1 Chemical analysis of PV

**FIGURE 1:**
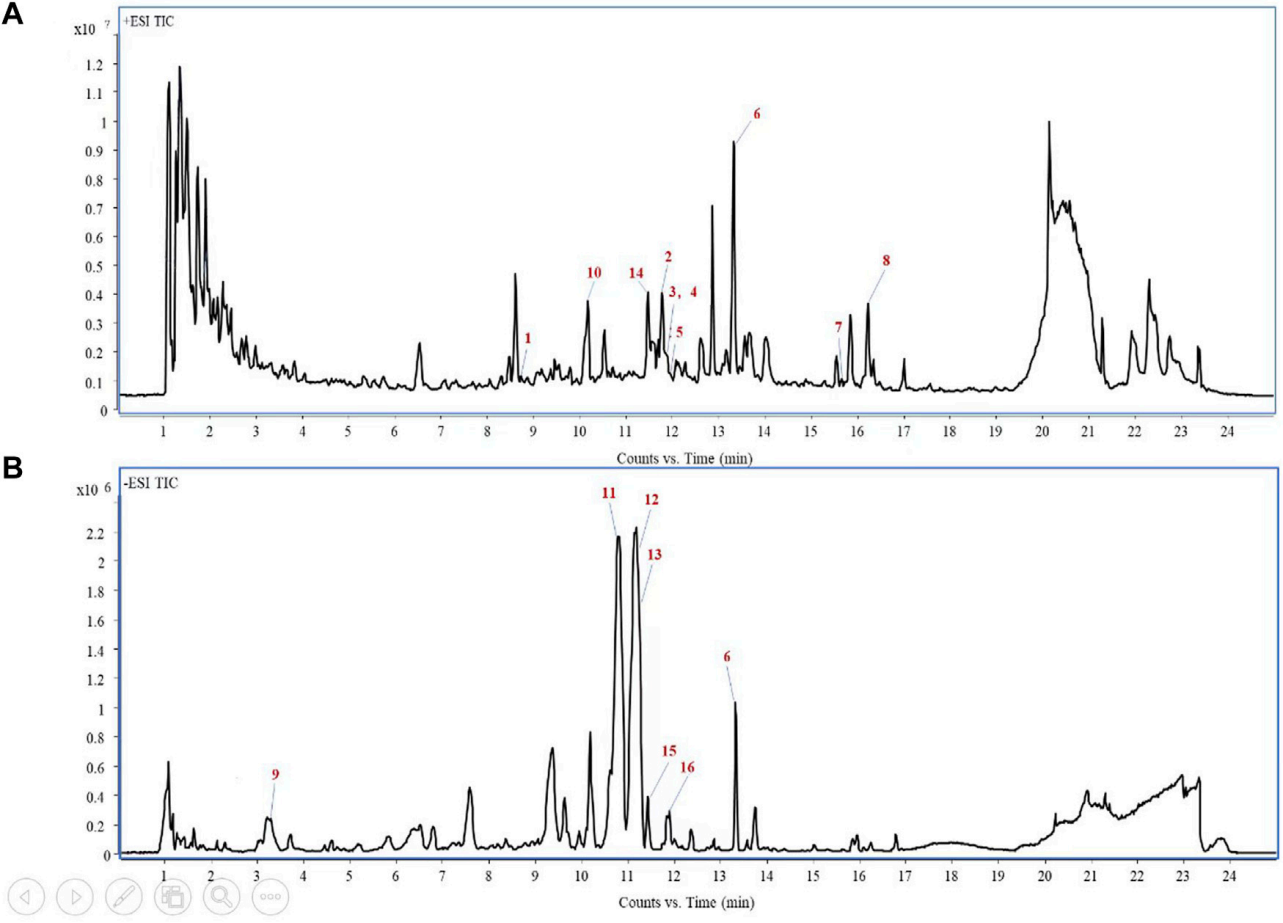
Total-ion chromatogram of PV extracts in the positive ion mode **(A)** and negative ion mode **(B)**.

PV was examined by UPLC QTOF-MS for its components to afford base peak chromatogram (Figure 1). The UPLC QTOF-MS result is shown in Table 1, which shows the retention times, molecular formula, and m/z. A total of 16 chemical compounds were identified (Figure 1; Table 1).

**TABLE 1:** Compound information.

Peak	Compound	Formula	Mass	Base peak	Precursor	*R*_*t*_ (min)
1	Umbelliferone	C_9_H_6_O_3_	162.0313	163.0386	+	8.761
2	Chicoric acid	C_22_H_18_O_12_	474.0783	497.0677	+	11.722
3	Ganoderic acid G	C_30_H_44_O_8_	532.302	555.2914	+	11.838
4	Atractylenolide III	C_15_H_20_O_3_	248.1406	249.148	+	11.822
5	Luteolin-3-D-glucuronide	C_21_H_18_O_12_	462.0786	463.086	+	11.987
6	19-Hydroxy-10-deacetylbaccatin lll	C_29_H_36_O_11_	560.2237	599.187	+	13.394
7	Isotoosendanin	C_30_H_38_O_11_	574.2395	597.2289	+	15.71
8	Gomisin N	C_23_H_28_O_6_	400.1876	423.1768	+	16.289
9	Cryptochlorogenic acid	C_16_H_18_O_9_	354.0963	353.0892	-	3.514
10	8-Methylretusin-7-O-glucopyranoside	C_23_H_24_O_10_	460.1371	459.1298	-	10.430
11	Tenuifoliside A	C_31_H_38_O_17_	682.2098	681.203	-	10.926
12	Asperulosidic acid	C_18_H_24_O_12_	432.1248	477.1232	-	11.108
13	Xylobiose	C_10_H_18_O_9_	282.094	327.0924	-	11.357
14	Galloyl paeoniflorin	C_30_H_32_O_15_	632.1727	677.1713	-	11.324
15	Kaempferol-3-O-glucorhamnoside	C_27_H_30_O_15_	594.1596	593.1525	-	11.439
16	Kaempferol 7-O-β-D-glucoside	C_21_H_20_O_11_	448.1011	447.0939	-	11.836

### 3.2 PV alleviates CCl_4_-induced acute liver injury

**FIGURE 2:**
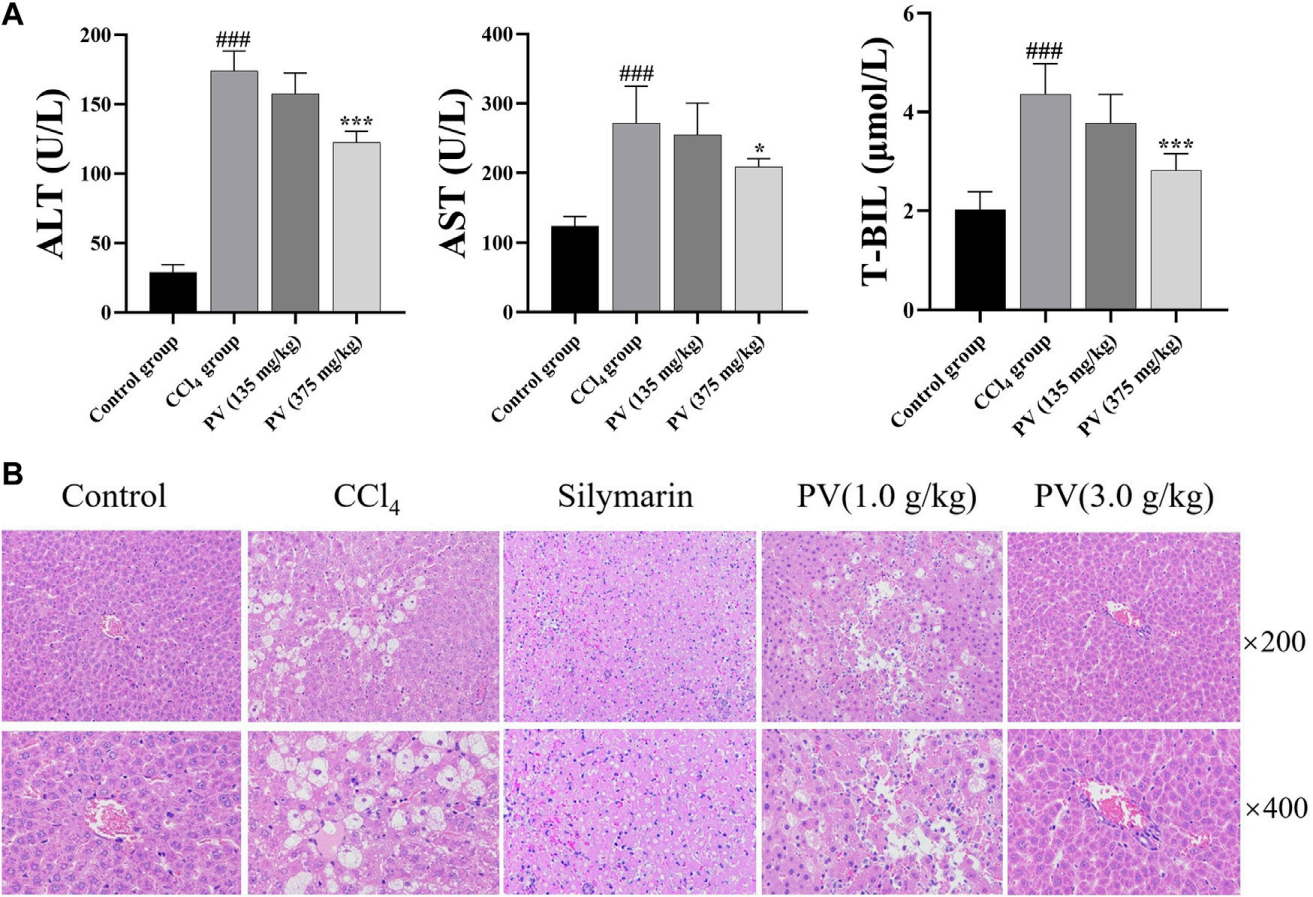
PV attenuates CCl_4_-induced liver injury of rats. **(A)** ALT, AST, and T-BIL levels in the serum. Values are the means ± SEM (n = 6). ^#^ *p* < 0.05 compared with the control group; *p < 0.05 compared with the CCl_4_ group. **(B)** H&E-stained liver section; silymarin (150 mg/kg).

We conducted biochemical and histological studies to assess the effectiveness of PV (125 and 375 mg/kg) in protecting the liver against injuries induced by CCl_4_ with silymarin (150 mg/kg) as the positive drug. Biochemical analysis showed that CCl_4_ administration increased the levels of AST, ALT, and T-BIL in the serum, whereas treatment with PV (125 and 375 mg/kg) showed a slight and potent significant reduction in these parameters, respectively (Figure 2A). Histologically, the model group showed potent inflammatory cell infiltration and resulted in injury to the structures of liver tissues, hepatocyte necrosis, and pseudolobules following CCl_4_ stimulation. On the other hand, PV reduced inflammatory cell infiltration, restored liver tissues and structures of hepatocytes, and improved the formation of pseudolobules (Figure 2B). As 375 mg/kg of PV possesses the most evident protective effect on the liver, the following mechanism study would adopt PV (375 mg/kg) to show the underlying mechanism of PV.

### 3.3 PV alleviates CCl_4_-induced cell apoptosis in the liver

**FIGURE 3:**
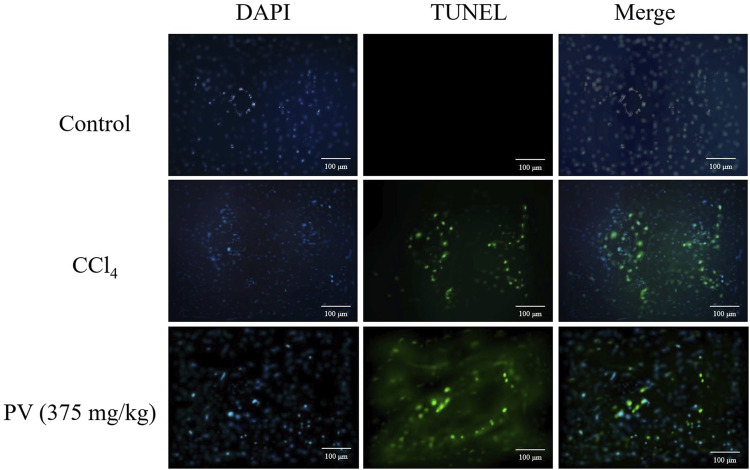
TUNEL-stained results of liver tissue showing that PV ameliorates apoptosis caused by CCl_4_ treatment.

TUNEL-stained liver sections were used to monitor apoptosis caused by CCl_4_. As a result, CCl_4_ induced potent apoptosis in liver tissues, whereas PV (375 mg/kg) significantly ameliorated CCl_4_-induced apoptosis in the liver tissue (Figure 3).

### 3.4 PV suppresses autophagy in HepG2 cells treated with CCl_4_

**FIGURE 4:**
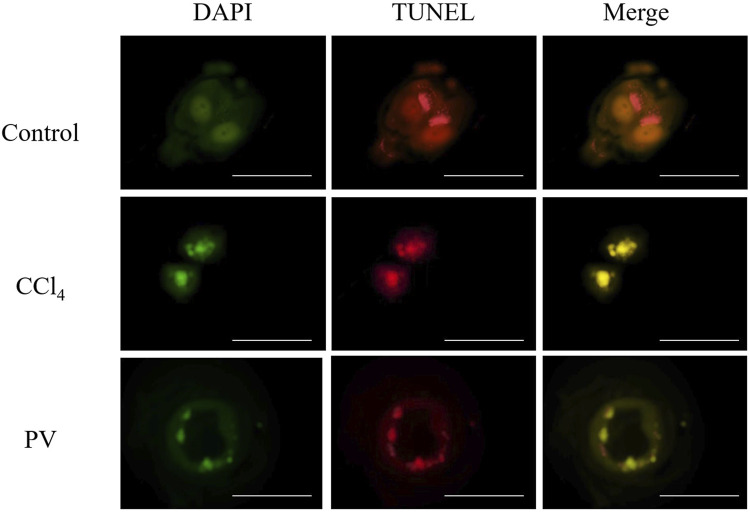
PV suppresses autophagy in HepG2 cells treated with CCl_4_. HepG2 cells were transiently transfected with RFP-GFP-LC3 vector. After 48 h, cells were treated with CCl_4_ at 20 mM or PV at 20 μg/mL for 12 h.

HepG2 cells were used to detect autophagy caused by CCl_4_. After 48-h transient transfection with RFP-GFP-LC3 vector, cells were treated with CCl_4_ at 20 mM or PV at 20 μg/mL for 12 h. Yellow color indicated the inhibition of autophagy flux caused by autophagosome–lysosome fusion impairment. The results showed that the CCl_4_ group showed the most yellow color, whereas the CCl_4_+PV group showed a relatively lighter yellow color than the CCl_4_ group (Figure 4).

### 3.5 PV restores bile acid levels in the serum of rats treated with CCl_4_

**FIGURE 5:**
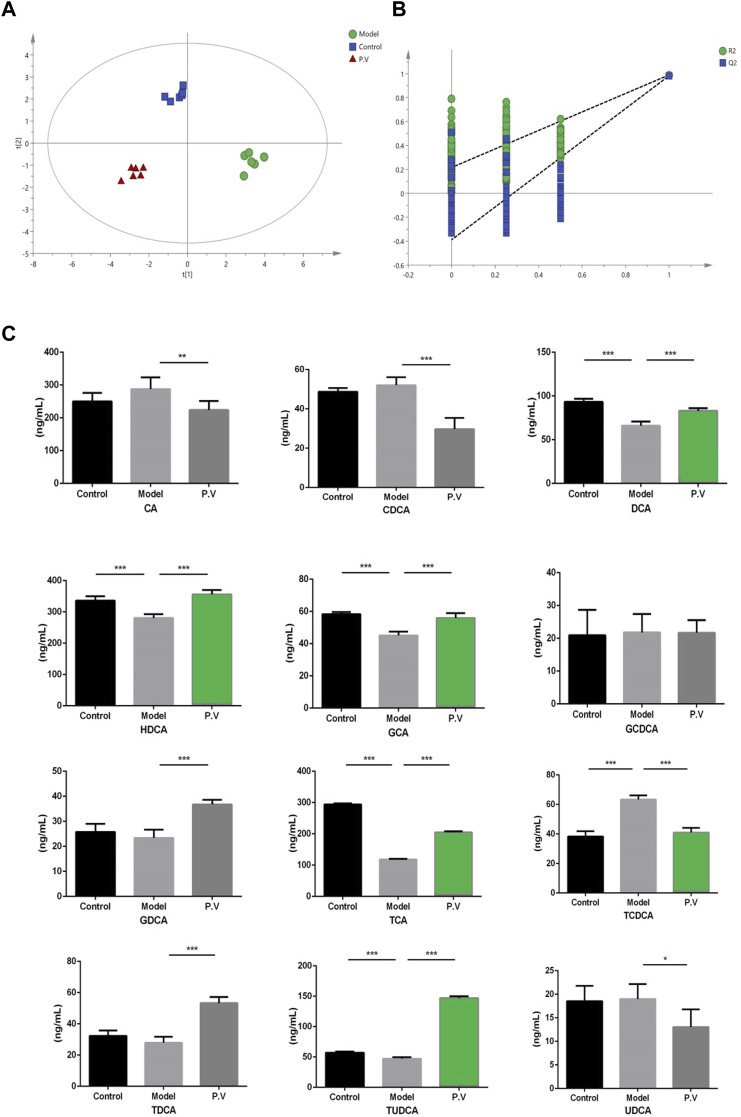
Effects of PV (375 mg/kg) on bile acid levels in the serum. Results of **(A)** PLS-DA score chart and P-test chart **(B)** of bile acid profile in the serum. **(C)** Levels of indicated bile acids in the serum detected by UPLC-QQQ MS. Values are the means ± SEM (n = 6). ^#^ *p* < 0.05 compared with the control group; *p < 0.05 compared with the model (CCl_4_) group. Model group: CCl_4_ group.

**FIGURE 6:**
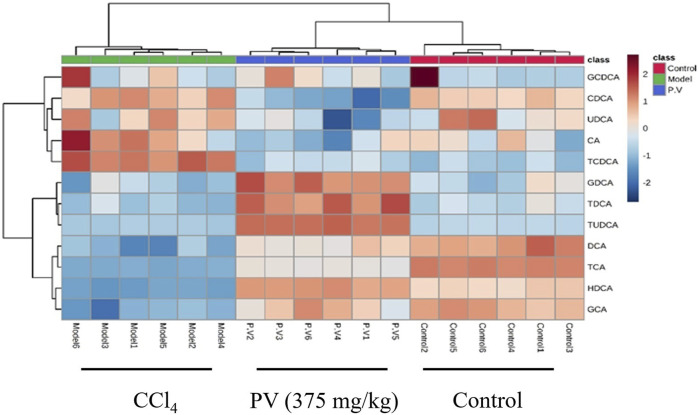
Heatmap analysis of serum bile acid. Heatmap results of serum bile acids described in Figure 5.

To investigate the effect of bile acids in the CCl_4_-driven injury in the liver, the bile acid profile was analyzed using the UPLC-QQQ MS method. As a result, the PLS-DA score chart and P-test chart of the bile acid profile in the serum indicated a significant difference in bile acid levels after CCl_4_ administration (Figures 5A and B). Specifically, the levels of six bile acids, including DCA, HDCA, GCA, TCA, TCDCA, and TUDCA, were significantly altered after CCl_4_ administration, and the level of all these six bile acids was restored by PV (375 mg/kg) treatment (Figures 5C and 6).

### 3.6 PV restores bile acid levels in the liver of rats treated with CCl_4_

**FIGURE 7:**
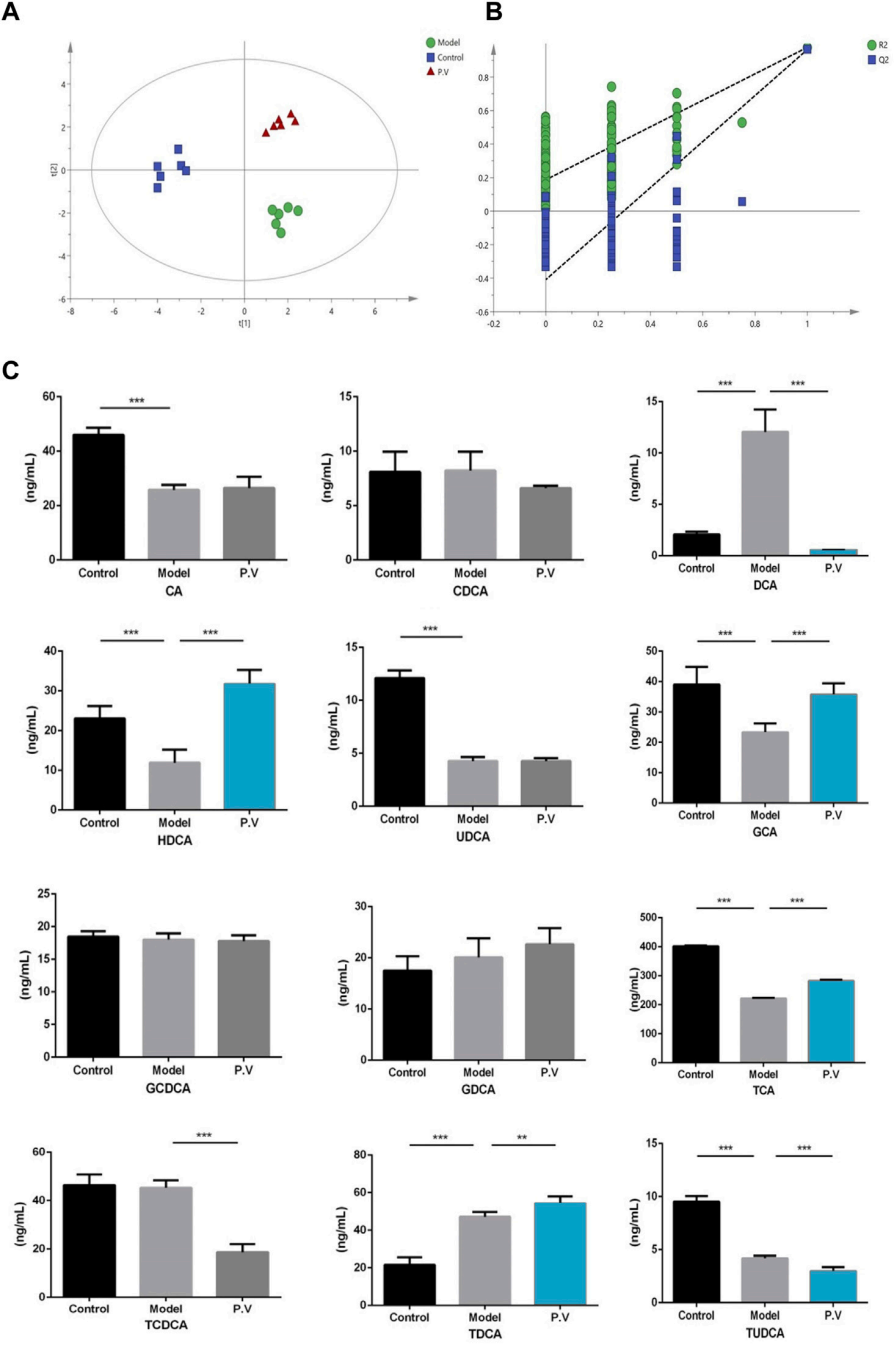
Effects of PV (375 mg/kg) on bile acid levels in the liver. Results of **(A)** PLS-DA score chart and P-test chart **(B)** of bile acid profile in the liver. **(C)** Levels of indicated bile acids in the liver detected by UPLC-QQQ MS. Values are the means ± SEM (n = 6). ^#^ *p* < 0.05 compared with the control group; *p < 0.05 compared with the model (CCl_4_) group. Model group: CCl_4_ group.

**FIGURE 8:**
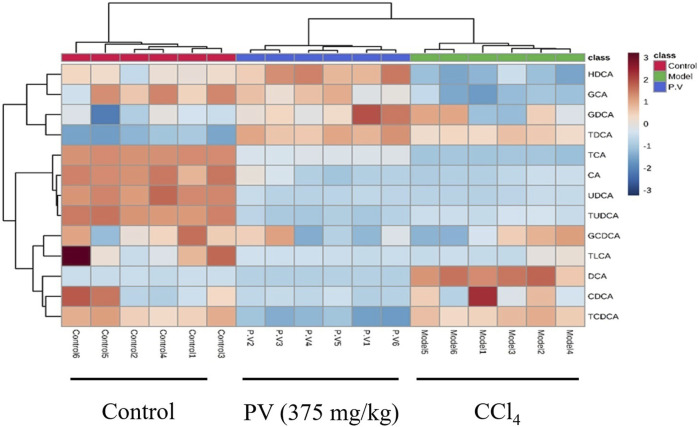
Heatmap analysis of liver bile acids.

To further confirm the results from the serum, the bile acid profile of the liver in rats treated with CCl_4_ was tested. Similar to what was observed in the serum, the levels of six bile acids (DCA, HDCA, GCA, TCA, TCDCA, and TUDCA) were significantly altered after CCl_4_ administration, and the level of all these six bile acids was restored by PV (375 mg/kg) treatment (Figures 7 and 8).

## 4 Discussion

Liver fibrosis is a multi-faceted process that develops in reaction to diverse forms of liver damage. Understanding the underlying causes and mechanisms of liver fibrosis is crucial for developing effective treatment strategies. This process is characterized by the accumulation of extracellular matrix (ECM) components such as collagen, which leads to the disruption of the normal liver structure and function. Liver fibrosis can progress through several stages, ranging from mild fibrosis to severe cirrhosis, which is characterized by the formation of regenerative nodules and the loss of normal liver function. In some cases, liver fibrosis can progress to hepatocellular carcinoma (HCC), whose mortality rate is high.

Efforts to address liver fibrosis are crucial for promoting public health and well-being because it can lead to significant morbidity and mortality. Patients with advanced liver fibrosis are at an increased risk of developing complications such as portal hypertension and ascites. Moreover, liver fibrosis is a major risk factor for HCC.

Carbon tetrachloride (CCl_4_) is a laboratory reagent that is widely used in liver-related studies because it is highly toxic and can cause liver lesions and fibrosis. In CCl_4_-treated rats, a significant increase in the lipid profile and oxidative stress marker was observed. At the same time, there was a dramatic decrease in HDL. Microscopic examination of the treated rats revealed fatty changes, inflammatory accumulation, injury in normal hepatocytes, collagen deposition, and the formation of fiber segmentations (Ebeid et al., 2015).

Previous research suggests that bile acids can cause direct harm to the liver by destroying liver cells due to their detergent cytolytic effects (Schölmerich et al., 1984). This indicates that toxic bile acids can kill liver cells when added bile acids are added to them at very low levels. However, in pathological conditions, the levels of toxic bile acids in the bloodstream and tissue rarely reach such submillimolar levels, which strongly suggests that it is possible that the liver cell death is not predominantly caused by the cytolytic properties. It was proposed that bile acids induce apoptosis in hepatocytes, which was supported by evidence of rat liver apoptosis. Nevertheless, these pieces of evidence imply that under pathophysiological circumstances, the toxicity may not be the primary reason for the injury caused by bile acids. Moreover, the bile acid profile in the CCl_4_-induced liver injury so far is known.

In this paper, we for the first time revealed that six bile acids, namely, DCA, HDCA, GCA, TCA, TCDCA, and TUDCA, were significantly altered after CCl_4_ administration, and the level of all these six bile acids can be restored by PV treatment (Figures 4–7). So far, the effect of DCA (Zhao et al., 2020), TCDCA (Fukumoto et al., 2002), and TUDCA (Sun et al., 2020) on liver injury has been investigated, whereas the role of HDCA, GCA, and TCA in liver injury is not known, which needs in-depth studies in the future. Second, in this paper, we found that the apoptosis and autophagy caused by CCl_4_ were ameliorated by PV treatment. Recently, it was reported that miR-217 could target NAT2 to promote apoptosis and autophagy simultaneously in a CCl_4_-induced rat model (Yang et al., 2019). Therefore, it is likely that PV might suppress miR-217 to inhibit apoptosis and autophagy caused by CCl_4_, which should be studied in future studies.

## Data Availability

The data presented in the study are deposited in the zenodo repository, available at https://zenodo.org/records/10895272.
