# Supplementary material for: Patrinia villosa (Thunb.) Juss alleviates CCL4-induced acute liver injury by restoring bile acid levels and inhibiting apoptosis/autophagy
Source: Front Pharmacol. 2024 May 22;15:1409971. doi: 10.3389/fphar.2024.1409971 (PMC11150553; doi:10.3389/fphar.2024.1409971)
Supplement: Supplementary file 1 [file Table1.DOCX]

Supplementary Material

*Patrinia villosa* Juss alleviates acute liver injury through restoring bile acids and inhibiting apoptosis/autophagy

Jifeng Ye^1^, Wei Liu^2, 3^, Qishu Hou^1^, Shu-qi Bai^4^, Zheng Xiang^2, 3^, Jiaqi Wang^2, 3*^, Liman Qiao^1*^

^1^ Department of Pharmacy, the Second Affiliated Hospital and Yuying Children's Hospital of Wenzhou Medical University, Wenzhou, China

^2^ School of Pharmaceutical Science, Liaoning University, Shenyang, China

^3^ Shenyang Key Laboratory for Causes and Drug Discovery of Chronic Diseases, Liaoning University, Shenyang, China

^4^ Liaoning Inspection, Examination & Certification Centre, Liaoning Province Product Quality Supervision and lnspection Institute, Shenyang, China

*** Correspondence:**Jiaqi Wang,
[albbwangjiaqi@163.com](mailto:albbwangjiaqi@163.com)

# Liman Qiao, [qiaoliman1980@163.com](mailto:qiaoliman1980@163.com)

**Table S1** Linear equation, linear regression equation, R^2^ and linear interval

| Comd. | Standard curves | R^2^ | Range |
| --- | --- | --- | --- |
| CA | y = 0.0442x - 0.9773 | 0.9930 | 1-1000 ng/mL |
| CDCA | y = 0.0161x - 0.1006 | 0.9846 | 1-1000 ng/mL |
| DCA | y = 0.0176x + 0.0459 | 0.9996 | 1-1000 ng/mL |
| HDCA | y = 0.0061x - 0.0461 | 0.9943 | 1-1000 ng/mL |
| GCA | y = 0.0014x - 0.0132 | 0.9976 | 1-1000 ng/mL |
| GCDCA | y = 0.0012x - 0.0201 | 0.9976 | 1-1000 ng/mL |
| GDCA | y = 0.001x - 0.0110 | 0.9923 | 1-1000 ng/mL |
| TCA | y = 0.0228x - 0.268 | 0.9982 | 1-1000 ng/mL |
| TCDCA | y = 0.0132x - 0.1291 | 0.9987 | 1-1000 ng/mL |
| TDCA | y = 0.0607x - 0.1885 | 0.9942 | 1-1000 ng/mL |
| TUDCA | y = 0.0294x + 0.0455 | 0.9921 | 1-1000 ng/mL |
| UDCA | y = 0.0069x - 0.0041 | 0.9932 | 1-1000 ng/mL |
| LCA | y = 0.0177x - 0.0259 | 0.9958 | 1-1000 ng/mL |
| TLCA | y = 0.0006x - 0.0038 | 0.9852 | 1-1000ng/mL |

**Table S2** Accuracy and precision of bile acids.

| Comd. | Conc. (ng/ml) | Accuracy  % | Precision% | Repatbility % | Stability % |
| --- | --- | --- | --- | --- | --- |
| CA | 250 | 100.37±5.31 | 5.29 | 4.69 | 0.41 |
|  | 500 | 98.26±6.24 | 6.35 | 4.56 | 1.69 |
|  | 1000 | 99.35±7.47 | 7.52 | 5.34 | 1.85 |
| CDCA | 250 | 98.62±6.48 | 6.57 | 6.45 | 2.78 |
|  | 500 | 94.58±8.36 | 8.84 | 2.87 | 3.80 |
|  | 1000 | 93.67±4.67 | 4.99 | 4.24 | 2.14 |
| DCA | 250 | 95.43±4.26 | 4.46 | 4.72 | 3.07 |
|  | 500 | 100.23±7.48 | 7.46 | 5.71 | 3.18 |
|  | 1000 | 97.34±8.47 | 8.70 | 4.56 | 1.09 |
| HDCA | 250 | 98.79±7.34 | 7.43 | 6.56 | 0.71 |
|  | 500 | 95.43±8.56 | 8.97 | 6.77 | 3.35 |
|  | 1000 | 100.34±8.49 | 8.46 | 3.45 | 4.00 |
| GCA | 250 | 99.78±9.23 | 9.25 | 4.55 | 2.98 |
|  | 500 | 100.15±9.35 | 9.34 | 6.00 | 1.73 |
|  | 1000 | 93.46±4.56 | 4.88 | 7.67 | 2.09 |
| GCDCA | 250 | 99.24±7.23 | 7.29 | 7.90 | 3.65 |
|  | 500 | 100.23±6.46 | 6.45 | 3.45 | 6.16 |
|  | 1000 | 100.09±9.34 | 9.33 | 7.89 | 7.36 |
| GDCA | 250 | 98.23±8.34 | 8.49 | 3.83 | 1.47 |
|  | 500 | 96.43±8.45 | 8.76 | 4.97 | 2.47 |
|  | 1000 | 97.23±6.34 | 6.52 | 5.17 | 5.22 |
| TCA | 250 | 94.89±6.45 | 6.80 | 5.78 | 1.45 |
|  | 500 | 92.78±5.49 | 5.92 | 7.89 | 0.89 |
|  | 1000 | 95.23±4.78 | 5.02 | 4.67 | 2.45 |
| TDCA | 250 | 98.34±7.48 | 7.61 | 6.88 | 0.41 |
|  | 500 | 99.56±7.65 | 7.68 | 7.34 | 1.69 |
|  | 1000 | 91.37±3.45 | 3.78 | 7.45 | 1.85 |
| TUDCA | 250 | 95.22±5.67 | 5.95 | 6.10 | 2.78 |
|  | 500 | 96.75±8.34 | 8.62 | 2.87 | 4.98 |
|  | 1000 | 97.56±6.45 | 6.61 | 4.24 | 3.75 |
| UDCA | 250 | 94.59±8.45 | 8.93 | 4.36 | 6.03 |
|  | 500 | 99.87±5.89 | 5.90 | 3.89 | 6.78 |
|  | 1000 | 97.65±3.45 | 3.53 | 5.34 | 5.47 |
| LCA | 250 | 98.34±7.45 | 7.58 | 3.55 | 0.67 |
|  | 500 | 99.28±8.43 | 8.49 | 5.67 | 3.34 |
|  | 1000 | 95.47±9.35 | 9.79 | 3.43 | 2.00 |
| TLCA | 250 | 98.46±4.66 | 4.73 | 3.56 | 1.73 |
|  | 500 | 97.47±4.27 | 4.38 | 4.34 | 2.64 |
|  | 1000 | 93.28±8.55 | 9.17 | 4.23 | 6.78 |
| TCDCA | 250 | 99.46±9.34 | 9.39 | 5.43 | 7.44 |
|  | 500 | 100.05±7.63 | 7.63 | 5.77 | 5.43 |
|  | 1000 | 96.49±8.54 | 8.85 | 4.67 | 3.23 |

**Table S3** The recovery of the bile acids.

| Comd. | Baseline (ng/ml) | Spiked (ng/ml) | Recovery % |
| --- | --- | --- | --- |
| CA | 1000 | 250 | 90.63±8.13 |
|  |  | 500 | 93.54±10.52 |
|  |  | 750 | 93.53±9.64 |
| CDCA | 1000 | 250 | 85.46±10.57 |
|  |  | 500 | 90.52±12.43 |
|  |  | 750 | 93.27±10.26 |
| DCA | 1000 | 250 | 89.44±11.15 |
|  |  | 500 | 90.46±6.54 |
|  |  | 750 | 91.72±10.19 |
| HDCA | 1000 | 250 | 87.04±13.23 |
|  |  | 500 | 89.42±7.12 |
|  |  | 750 | 92.16±8.25 |
| GCA | 1000 | 250 | 85.48±7.21 |
|  |  | 500 | 87.12±8.42 |
|  |  | 750 | 86.20±6.13 |
| GCDCA | 1000 | 250 | 92.32±7.36 |
|  |  | 500 | 93.64±6.46 |
|  |  | 750 | 93.43±7.15 |
| GDCA | 1000 | 250 | 90.47±6.12 |
|  |  | 500 | 92.26±9.62 |
|  |  | 750 | 91.44±9.18 |
| TCA | 1000 | 250 | 89.19±8.33 |
|  |  | 500 | 91.23±8.01 |
|  |  | 750 | 90.34±6.81 |
| TDCA | 1000 | 250 | 91.22±10.08 |
|  |  | 500 | 92.47±8.15 |
|  |  | 750 | 90.89±7.16 |
| TUDCA | 1000 | 250 | 89.23±8.37 |
|  |  | 500 | 92.77±8.32 |
|  |  | 750 | 90.32±10.28 |
| UDCA | 1000 | 250 | 92.33±6.73 |
|  |  | 500 | 90.77±4.27 |
|  |  | 750 | 89.26±8.39 |
| LCA | 1000 | 250 | 89.47±7.32 |
|  |  | 500 | 93.27±10.92 |
|  |  | 750 | 93.45±8.74 |
| TLCA | 1000 | 250 | 87.27±10.77 |
|  |  | 500 | 90.84±9.45 |
|  |  | 750 | 90.23±8.34 |
| TCDCA | 1000 | 250 | 84.23±7.37 |
|  |  | 500 | 86.24±9.45 |
|  |  | 750 | 87.56±10.34 |
